# Comparative Analysis of Chemical Composition and Antibacterial Activity of Essential Oils from Five Varieties of *Lavender* Extracted via Supercritical Fluid Extraction

**DOI:** 10.3390/molecules30020217

**Published:** 2025-01-07

**Authors:** Lijing Lin, Zhencheng Lv, Meiyu Wang, Ankang Kan, Songling Zou, Bin Wu, Limin Guo, Salamet Edirs, Jiameng Liu, Lin Zhu

**Affiliations:** 1 Hainan Key Laboratory of Storage and Processing of Fruits and Vegetables, Agricultural Products Processing Research Institute, Chinese Academy of Tropical Agricultural Sciences, Zhanjiang 524001, China; linlijing0763@163.com (L.L.); 202330110016@stu.shmtu.edu.cn (M.W.); 2 School of life Sciences, Huizhou University, Huizhou 516007, China; szsky@hzu.edu.cn (Z.L.); 18038660656@163.com (S.Z.); 3 Merchant Marine College, Shanghai Maritime University, Shanghai 201306, China; ankang0537@126.com; 4 Institute of Agro-Production Storage and Processing, Xinjiang Academy of Agricultural Sciences, Urumqi 830091, China; xjuwubin0320@sina.com (B.W.); guolm_xj@163.com (L.G.); salamet619@163.com (S.E.)

**Keywords:** antibacterial activity, essential oil, *Lavender*, supercritical fluid extraction, volatile components

## Abstract

This study aimed to determine the chemical composition of five *Lavender* essential oils (LEOs) using the gas chromatography–mass spectroscopy technique and to assess their antibacterial activity against four marine *Vibrio* species, including *Shewanella algae*, *Shewanella maridflavi*, *Vibrio harveyi*, and *Vibrio alginolyticus*. Sensitivity tests were performed using the disk diffusion and serial dilution methods. The results showed that all five LEOs exhibited antibacterial activity against the four tested marine *Vibrio* species. The antibacterial activities of all five LEOs were above moderate sensitivity. The five LEOs from French blue, space blue, eye-catching, and true *Lavender* showed high sensitivity, particularly against *Shewanella maridflavi*. The compounds of LEOs from different varieties of *Lavender* were similar and mainly comprised linalool, linalyl acetate, eucalyptol, and isoborneol. Different varieties of LEOs possessed unique components besides common components, and the percentage of each one was different, which led to different fragrance loads. The major fragrances were lily of the valley, an aromatic compound fragrance, and an herbal fragrance. The antibacterial activity of LEO from eye-catching *Lavender* was better than that of others, which could provide a reference for its application in the prevention and control of marine *Vibrio* spp. and the development of antibacterial products.

## 1. Introduction

Essential oils, compounds extracted from plants, are volatile and aromatic liquids. The unique aromatic compounds give each essential oil its distinctive essence. Essential oils can retard or inhibit the growth of many bacteria and fungi [1]. For example, tea tree essential oil and *thyme* essential oil showed significant antibacterial activity against *Staphylococcus aureus*. *Cinnamon* essential oil and *clove* essential oil have inhibitory effects on *Escherichia coli*. *Rosemary* essential oil and *Lavender* essential oil show antibacterial effect on *Pseudomonas aeruginosa* [2]. *Lindera subumbelliflora Kosterm* essential oil has antifungal activity against *Candida albicans* [3]. *Oregano* essential oil and *thyme* essential oil have inhibitory effects on *Aspergillus*, while lemon grass essential oil and cinnamon essential oil have antifungal effects on *Penicillium* [4]. These oils are composed of various aldehydes, phenols, alcohols, and other compounds, mainly terpenes and phenylpropanoids, with the former being the principal constituent [5]. Essential oils were initially used as fragrances. As research progressed, these oils were identified to possess multiple biological activities. Currently, they are used in traditional Chinese medicine compound preparations, aromatherapy agents, and insect repellents and have widespread applications in medicine, aquaculture, food, cosmetics, etc. [6]. Essential oils can be used as anesthetics, sedatives, and substitutes for synthetic drugs. Nalan Ozgur Yigit [7] found that *Lavender* and laurel essential oil had remarkable anesthetic effects on rainbow trout. The induction time of *Lavender* essential oil at 200 mg/L and laurel essential oil at 400 mg/L was 258.0 s and 189.5 s, respectively, and the recovery time was 41 s and 129.5 s. Histopathological evaluation showed no abnormalities in the gill, liver, and kidney. Soon-Il Kim [8] found that plant essential oil has the potential to become a new pesticide. Bakiaydn [9] also found that Lavender essential oil as a natural anesthetic may be a more environmentally friendly, cost-effective, and safer product than synthetic drugs. Essential oil can be used as a new antibacterial substance. Temitayo Margaret Omoyeni [10] uses freeze-dried polyvinyl alcohol (PVA)/Arabic gum (GA) to synthesize hydrogel, in which 0.2 mL of black Vitex negundo oil has an obvious inhibitory effect on Staphylococcus aureus and Bacillus subtilis and can be used for wound healing and other biomedical applications. With people’s quality of life continuously improving and the government’s increasing emphasis on sustainable development [11], plant essential oils can meet the requirements of being healthy, green, environmentally friendly, and pollution-free in their applications.

*Lavender* (*Lavandula angustifolia Mill.*) is a perennial subshrub plant belonging to the *Lamiaceae* family and the *Lavandula* genus and is native to the Mediterranean coast. *Lavender* essential oil (LEO) is known for its calming effect on the nerves [12], pain relief, and the treatment of neurological diseases. LEO is light yellow, extracted from *Lavender*, and has an aromatic fragrance. Studies have reported that LEO has a broad-spectrum antibacterial ability [13]. At present, GC-MS can be used to analyze the components of essential oil and study its antibacterial activity. Othman El Faqer [14] studied the antibacterial potential of *Allium sativum* essential oil. By GC/MS analysis, it was found that diallyl disulfide was the main component, and *Allium sativum* essential oil showed strong activity against methicillin-resistant Staphylococcus aureus. Gunja Sah [15] studied and analyzed the chemical constituents and biological activities of *Mentha longifolia*. By GC-MS analysis, it is confirmed that the main components are piperitone oxide and cis-piperitone oxide. The essential oil showed remarkable nematicidal activity, antioxidant activity (through DPPH and H_2_O_2_ scavenging experiments), and antibacterial activity against a variety of bacteria and fungi. *Salvia dumetorum* essential oil was extracted by Yana K. Levaya [16] by steam distillation, and its chemical constituents were analyzed by GC-MS, among which sesquiterpenes accounted for 54.15%. *Salvia dumetorum* essential oil showed strong antibacterial activity against Staphylococcus aureus and Bacillus subtilis and inhibited the biofilm formation of Streptococcus mutans on 1% sucrose medium.

The genus *Vibrio* (*Vibrio* spp.) comprises Gram-negative bacteria that are widely distributed in bays, coastal waters, open oceans, sediments, and marine environments [17]. Those organisms are opportunistic pathogens. Marine fisheries often suffer from vibriosis caused by *Vibrio* infections, resulting in substantial economic losses. Furthermore, this organism exerts a negative effect on the sustainable development of the marine ecological environment [18]. In addition, *Vibrio* can infect humans through the food chain via contaminated seafood, posing a severe threat to human health [19]. Currently, there are few reports on the antibacterial activity of essential oils against marine *Vibrio*.

Five varieties of *Lavender* were selected in this study, as follows: French blue *Lavender* (*Lavandula stoechas*), Xinjiang Ili 701 *Lavender*, space blue *Lavender* (*Lavandula stoechas*), eye-catching *Lavender* (*Lavandula hybrida*), and true *Lavender* (*Lavandula angustifolia*). Four bacterial strains were tested in this study, namely *Shewanella maridflavi*, *Shewanella algae*, *Vibrio alginolyticus*, and *Vibrio harveyi*, to investigate the antibacterial activity of the five LEOs. The main components of the essential oils were qualitatively and quantitatively analyzed using gas chromatography–mass spectroscopy (GC–MS). Moreover, their aroma characteristics were studied to provide a scientific basis for the development and utilization of essential oils in the aquaculture industry.

## 2. Results and Discussion

### 2.1. Supercritical Fluid Extraction of LEO

The results of the supercritical fluid extraction of LEOs are shown in Table 1. The essential oils obtained by supercritical fluid extraction were yellow liquids, with extraction yields ranging from 3.72% to 4.17%. Eye-catching *Lavender* had the highest extraction yield of 4.17 ± 0.11%. In terms of color, the essential oils were basically yellow, with true *Lavender* having a lighter color.

### 2.2. Analysis of Antibacterial Activity

#### 2.2.1. Antibacterial Activity of Essential Oils (Disk Diffusion Method)

**Figure 1:**
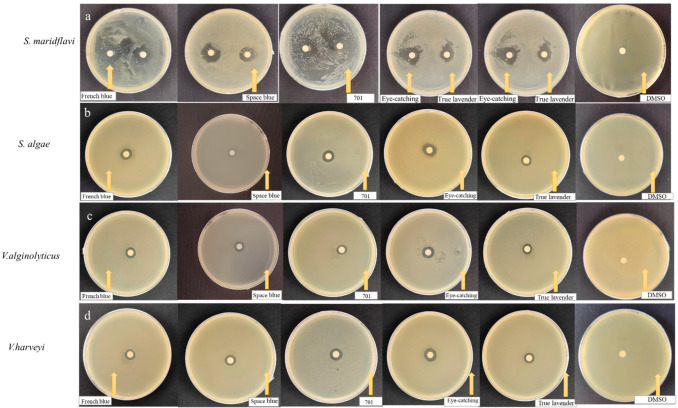
(**a**) Bacteriostatic effect of five *Lavender* essential oils against *Shewanella maridflavi*. (**b**) Inhibition zone effect of five LEOs against *Shewanella algae*. (**c**) Inhibition zone effect of five LEOs against *Vibrio alginolyticus*. (**d**) Inhibition zone effect of five LEOs against *Vibrio harveyi*.

The bacteriostatic effect of LEO against *S. maridflavi* is shown in Figure 1a. The sensitivity levels and diameters of the inhibition zones were as follows: eye-catching LEO, highly sensitive (17.17 mm) > French blue LEO, highly sensitive (16.00 mm) > true LEO, highly sensitive (15.50 mm) > space blue LEO, highly sensitive (15.33 mm) > 701 LEO, moderately sensitive (12.5 mm).

The bacteriostatic effect of LEOs against *S. algae* is shown in Figure 1b. The sensitivity levels and diameters of the inhibition zones were as follows: French blue LEO, moderately sensitive (12.75 mm) > eye-catching LEO, moderately sensitive (12.58 mm) > 701 LEO, moderately sensitive (12 mm) > space blue LEO, moderately sensitive (11.83 mm) > true LEO, moderately sensitive (10.83 mm).

The bacteriostatic effect of LEOs against *V. alginolyticus* is shown in Figure 1c. The sensitivity levels and diameters of the inhibition zones were as follows: eye-catching LEO, moderately sensitive (14.83 mm) > French blue LEO, moderately sensitive (13.67 mm) > space blue LEO, moderately sensitive (12.33 mm) > 701 LEO, moderately sensitive (12.33 mm) > true *Lavender* LEO, moderately sensitive (12 mm).

The bacteriostatic effect of LEOs against *V. harveyi* is shown in Figure 1d. The sensitivity levels and diameters of the inhibition zones were as follows: space blue LEO, moderately sensitive (13.83 mm) > eye-catching LEO, moderately sensitive (13.75 mm) > true LEO, moderately sensitive (13.17 mm) > 701 LEO, moderately sensitive (11 mm) > French blue LEO, moderately sensitive (10.83 mm).

#### 2.2.2. Results of Gradient Dilution Filter Paper Disk Method for LEO

According to the data in Table 2, the bacteriostatic effects of the five LEOs all reached moderate sensitivity or above. Notably, when *Shewanella maridflavi* was used as the test strain, the essential oils of French blue *Lavender* (16 mm), space blue lave *Lavender* (15.33 mm), eye-catching *Lavender* (17.17 mm), and true *Lavender* (15.5 mm) all reached the highly sensitive standard. Overall, the essential oil of eye-catching *Lavender* demonstrated good activity against *Shewanella maridflavi*, *Shewanella algae*, *Vibrio alginolyticus*, and *Vibrio harveyi*.

### 2.3. Volatile Components of Essential Oils

**Figure 2:**
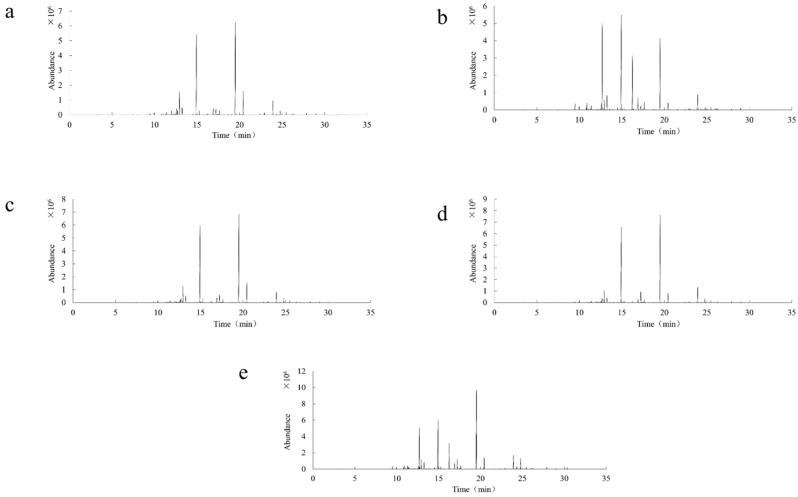
Total ion chromatogram of volatile components in LEOs ((**a**) French blue LEO; (**b**) 701 LEO; (**c**) space blue LEO; (**d**) eye-catching LEO; (**e**) true LEO).

The volatile components of French blue LEO were analyzed under the aforementioned GC–MS conditions. The total ion chromatogram of the volatile components is shown in Figure 2a. Through NIST/WILEY mass spectral library searching and the area normalization method, 29 compounds were identified from the volatile substances of French blue LEO, accounting for 96.33% of the total volatile components, as can be seen from Table 3, including 15 terpenes (15.9%), 7 alcohols (40.5%), 2 ketones (0.69%), and 5 esters (38.93%). The compounds with higher contents were linalool (32.72%), linalyl acetate (31.18%), eucalyptol (1.27%), trans-*β*-ocimene (4.72%), *β*-ocimene (2.02%), 1-octen-3-yl-acetate (1.14%), isoborneol (2.26%), terpinene-4-ol (2.56%), α-terpineol (1.17%), lavandulyl acetate (6.17%), β-caryophyllene (3.81%), and (E)-*β*-famesene (1.41%). These were the main components of French blue LEO, with compounds having a relative content > 1% accounting for 90.43%.

The volatile components of 701 LEO were analyzed under the aforementioned GC–MS conditions. The total ion chromatogram of the volatile components is depicted in Figure 2b. Through NIST/WILEY mass spectral library searching and the area normalization method, 29 volatile substances were identified from 701 LEO (99.14%), as can be seen from Table 4, including 16 terpenes (14.64%), 7 alcohols (44.30%), 2 ketones (0.6%), and 5 esters (38.07%). The compounds with higher contents were linalool (35.38%) and linalyl acetate (33.91%), followed by eucalyptol (1.51%), trans-β-ocimene (3.67%), β-ocimene (1.49%), isoborneol (1.94%), 4-carvomenthenol (3.71%), α-terpineol (1.05%), lavandulyl acetate (3.15%), β-caryophyllene (5.88%), and *(E)*-*β*-famesene (1.34%). These were the main components of 701 LEO, with compounds having a relative content > 1%, accounting for 93.03%.

The volatile components of space blue LEO were analyzed under the aforementioned GC–MS conditions. The total ion chromatogram of the volatile components is shown in Figure 2c. Through NIST/WILEY mass spectral library searching and the area normalization method, 33 compounds were identified from the volatile substances of space blue LEO, accounting for 99.29% of the total volatile components, as can be seen from Table 5, including 16 terpenes (20.49%), 8 alcohols (39.21%), 2 ketones (0.75%), and 7 esters (38.84%). The compounds with higher contents were linalool (31%) and linalyl acetate (30.27%), followed by 3-carene (1.17%), D-limonene (1.84%), eucalyptol (1.34%), trans-β-ocimene (4.75%), β-ocimene (1.96%), 1-octenyl-3-acetate (1.04%), isoborneol (2.87%), terpinene-4-ol (1.68%), α-terpineol (1.46%), lavandulyl acetate (6.51%), β-caryophyllene (5.04%), and (E)-*β*-Famesene (1.31%). These were the main components of space blue LEO, with compounds having a relative content > 1% accounting for 92.24%.

The volatile components of eye-catching LEO were analyzed under the aforementioned GC–MS conditions. The total ion chromatogram of the volatile components is shown in Figure 2d. Through NIST/WILEY mass spectral library searching and the area normalization method, 32 compounds were identified from the volatile substances of LEO, accounting for 99.78% of the total volatile components, as can be seen from Table 6, including 18 terpenes (17.92%), 7 alcohols (52.03%), 2 ketones (12.07%), and 5 esters (17.76%). The compounds with higher contents were linalool (27.51%), linalyl acetate (15.58%), eucalyptol (18.16%), and camphor (11.87%), followed by *α*-pinene (1.18%), *β*-pinene (1.35%), D-limonene (1.57%), trans-β-ocimene (1.85%), β-ocimene (2.88%), isoborneol (3.29%), α-terpineol (1.66%), lavandulyl acetate (1.46%), and β-caryophyllene (3.92%). These were the main components of eye-catching LEO, with compounds having a relative content > 1% accounting for 92.28%.

The volatile components of true LEO were analyzed under the aforementioned GC-MS conditions. The total ion chromatogram of the volatile components is shown in Figure 2e. Through NIST/WILEY mass spectral library searching and the area normalization method, 27 compounds were identified from the volatile substances of true LEO, accounting for 100% of the total volatile components, as can be seen from Table 7, including 10 terpenes (18.12%), 9 alcohols (33.74%), 4 ketones (2.34%), and 4 esters (46.05%). The compounds with higher contents were linalyl acetate (40.81%) and linalol (24.6%), followed by 3-octanone (1.17%), trans-*β*-ocimene (3.18%), *β*-ocimene (1.57%), isoborneol (1.63%), terpinene-4-ol (3.7%), lavandulyl acetate (4.14%), *β*-caryophyllene (5.83%), trans-2-hydroxycinnamic acid (1.62%), and (E)-*β*-Famesene (4.09%), which were the main components of true LEO. Compounds with a relative content > 1% accounted for 92.34%.

### 2.4. Aroma Analysis of Essential Oils

**Figure 3:**
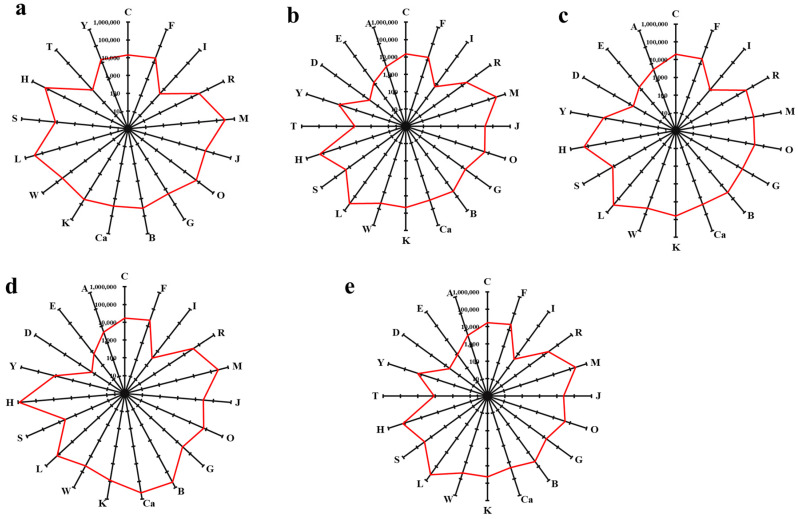
Radar chart of aroma distribution for each essential oil. Note: (**a**) French blue *Lavender*; (**b**) 701 *Lavender*; (**c**) space blue *Lavender*; (**d**) eye-catching *Lavender*; (**e**) true *Lavender*; each letter represents a different aroma: A—fatty; B—cool; C—citrus; D—frankincense; E—food; F—fruity; G—green; H—herbaceous; I—iris; J—jasmine; K—pine; L—aromatic compounds; M—lily of the valley; N—narcotic; O—orchid; P—phenolic; R—rose; S—spicy; T—toasted; Ve—vegetable; W—woody; Y—earthy; Z—organic solvent; Ca—camphor.

Volatile aromatic components are the material basis for the aroma of essential oils. By combining the ABC quantitative values of the odor of each essential oil substance with its relative content, a radar chart of the aroma distribution (Figure 3) can be drawn to visually express the overall aroma of different essential oils.

The aroma distribution radar chart of French blue LEO (Figure 3a) showed that this essential oil covered 17 aroma types. Of these, the aroma with the highest load was the lily of the valley, followed by aromatic compounds and orchids. Additionally, citrus, frankincense, fruity, iris, rose, jasmine, green, cool, camphor, pine, woody, spicy, herbaceous, and toasted aromas displayed high loads and constituted the chief aroma of French blue LEO.

701 LEO (Figure 3b) covered 20 aroma types. Of these, the aromas with the highest loads were the lily of the valley and aromatic compounds, followed by herbaceous. Moreover, fatty, cool, citrus, frankincense, food, fruity, green, iris, jasmine, pine, orchid, rose, spicy, toasted, woody, earthy, and camphor aromas exhibited high loads and constituted the primary aroma of 701 LEO.

Space blue LEO (Figure 3c) encompassed 18 aroma types, of which the one with the highest load was aromatic compounds, followed by herbaceous. Furthermore, fatty, cool, citrus, frankincense, food, fruity, green, iris, pine, lily of the valley, orchid, rose, spicy, woody, earthy, and camphor aromas had high loads and comprised the main aroma of space blue LEO.

Eye-catching LEO (Figure 3d) covered 19 aroma types, of which the one with the highest load was herbaceous, followed by cool and lily of the valley. Moreover, citrus, frankincense, food, fruity, green, herbaceous, iris, jasmine, pine, aromatic compounds, orchid, rose, spicy, woody, earthy, and camphor aromas exhibited high loads and constituted the primary aroma of eye-catching LEO.

True LEO (Figure 3e) covered 20 aroma types. Of these, the aroma with the highest load was aromatic compounds, followed by lily of the valley and herbaceous. In addition, fatty, cool, citrus, frankincense, food, fruity, green, iris, jasmine, pine, orchid, rose, spicy, toasted, woody, earthy, and camphor aromas had high loads and comprised the main aroma of true LEO.

### 2.5. Discussion

The essential oils of the five *Lavender* varieties, including French blue, caused moderate-to-high sensitivity in the four marine *Vibrio* strains, including *Shewanella maridflavi*. GC–MS findings indicated that the major components of the essential oils from all *Lavender* varieties were linalool and linalyl acetate. These observations agree with those from a previous report [35]. Linalool has been reported to exhibit bacteriostatic activity against *Pseudomonas putida* [36]. Several studies have shown that the antibacterial activity increases when the contents of linalool and linalyl acetate in LEOs are increased, which suggests that these two compounds are the main substances responsible for the antibacterial activity of LEOs. Linalool and linalyl acetate can play an antibacterial role by destroying the cell membrane of microorganisms, increasing the permeability of the membrane, leading to the leakage of important components of cells, thus causing cell death and inhibiting the synthesis of essential biological macromolecules (such as RNA, DNA, protein, and polysaccharides), further enhancing their antibacterial properties. At the same time, the stability of linalyl acetate in the process of thermal- and photo-oxidation affects its antibacterial efficacy, and proper treatment can enhance its activity [37,38]. In addition, linalool shows significant antifungal activity, which may interfere with the metabolic processes of fungi by inhibiting the activities of some key enzymes, destroy the biofilm structure of fungi, and inhibiting the formation and maturity of their biofilm [39]. The antibacterial effect of linalyl acetate may be partly due to the disturbance of the lipid part of the microbial plasma membrane, which leads to a change in membrane permeability and the leakage of intracellular substances. In addition, these compounds may penetrate the cell membrane and interact with key targets in the cell, thus exerting antibacterial activity [40]. LEOs exhibited good antimicrobial activities against *E. coli* and *S. aureus* at concentrations > 2000 ppm [41]. GC–MS data indicated that the essential oils of the five *Lavender* varieties used in this study contained approximately 30% linalool and linalyl acetate, which agrees with the moderate-to-high sensitivity demonstrated in the antibacterial activity assays. For the antibacterial mechanism of *Lavender* essential oil, we can deeply study its effects on the microbial cell membrane, protein synthesis, and nucleic acid structure, so as to better understand its mechanism.

The essential oil extracted from different lavender varieties produces distinct compounds. The unique compound found in 701 LEO is 4-Carvomenthenol. For space blue LEO, the distinctive compounds include Nerol, Hexyl 2-methylbutyrate, Bornyl acetate, and Neryl acetate. Eye-catching LEO is characterized by α-Sabine, γ-Terpinene, (E)-Hexyl 2-methylbut-2-enoate, and Neryl propionate. Lastly, the unique compounds identified in true LEO are 3-Octanol, trans-2-Hydroxycinnamic acid, caryophyllene oxide, and Herniarin.

The essential oil samples were characterized based on sensory attributes, such as sweet, floral, woody, camphor, fruity, and herb notes [41]. According to the aroma radar charts, different *Lavender* varieties exhibited different aroma types. Five substances, namely caryophyllene, geraniol acetate, geraniol, linalool, and linalyl acetate, have been shown to be the key compounds that differentiate the aromatic profiles of LEOs [42]. Linalool contributed the most to the lily of the valley aroma [43], linalyl acetate contributes the most to the aromatic compounds aroma [44], and isoborneol contributes the most to the herbaceous aroma. The essential oil of French blue *Lavender* had the highest aroma load of lily of the valley, followed by aromatic compounds and orchid; 701 LEO had the highest aroma load of lily of the valley and aromatic compounds, followed by herbaceous; space blue LEO had the highest aroma load of aromatic compounds, followed by herbaceous; eye-catching LEO had the highest aroma load of lily of the valley and aromatic compounds, followed by herbaceous; true LEO has the highest aroma load of aromatic compounds, followed by lily of the valley and herbaceous. All *Lavender* varieties displayed either the lily of the valley aroma from linalool or the aromatic compounds aroma from linalyl acetate. These findings imply that diversified *Lavender* varieties, including French blue, 701, space blue, eye-catching, and true *Lavender*, share common aroma components of *Lavender*. Nonetheless, each variety also possesses its unique aroma.

## 3. Materials and Methods

### 3.1. Materials

*Lavender* flowers: eye-catching *Lavender*, space blue *Lavender*, French blue *Lavender*, 701 *Lavender*, and true *Lavender* were all collected from Yining County, Ili Kazakh Autonomous Prefecture, Xinjiang (Latitude: 43.9779° N; Longitude: 81.5296° E).

Bacterial strains: *Shewanella algae* and *Shewanella maridflavi* were isolated from *Bahaba taipingensis*; *Vibrio harveyi* and *Vibrio alginolyticus* were isolated from *Ostrea gigas*. All strains were marine Vibrio provided by the Marine Products Research Laboratory of Huizhou University.

Dimethyl sulfoxide (DMSO) (Beijing Solarbio Science & Technology Co., Ltd., Beijing, China), marine culture medium, Luria Bertani Broth (LB broth), LB agar, agar, and peptone (Shanghai Acmec Biochemical Technology Co., Ltd., Shanghai, China) were used.

### 3.2. Instruments and Equipment

An HA121-50-2 Supercritical extraction device (Jiangsu Nantong Hua’an Supercritical Extraction Co., Ltd., Nantong, China); 7890B-5977A GC-MS (Agilent, Santa Clara, CA, USA), Shimadzu UV-2550 UV-visible spectrophotometer (Shimadzu (China) Co., Ltd., Shanghai, China), HP-5MS chromatographic column (30 m × 250 μm × 0.25 μm) (Agilent, USA), and Synergy H1 multi-function microplate detector (BioTek Instruments, Inc., Charlotte, VT, USA) were used.

### 3.3. Methods

#### 3.3.1. Extraction of LEO

*Lavender* flowers were accurately weighed (500 g) and placed into an extraction vessel (18 MPa, 40 °C, 2 h). The obtained oil was released from the separation vessel to obtain the essential oil. The extraction yield was finally calculated using the following formula:

$$\mathrm{Extraction}\;\mathrm{yield}\;\left(\%\right)=\left[\mathrm{Mass}\;\mathrm{of}\;\mathrm{essential}\;\mathrm{oil}\;/\;\mathrm{Mass}\;\mathrm{of}\;\mathrm{raw}\;\mathrm{material}\right]\times \;100$$

#### 3.3.2. Antibacterial Activity of LEOs

##### Preparation of Bacterial Suspensions

Experimental strains were stored in a −80 °C refrigerator. Each time, 2 μL of bacterial strains were inoculated into a liquid marine culture medium. The inoculation was performed in a laminar flow cabinet. After inoculation, the cultures were shaken and incubated at 35 °C for 10 h in an incubator shaker. Subsequently, the optical density (OD) values were measured at 600 nm using a spectrophotometer [45]. The optimal OD value range for the bacterial suspensions was 0.8–1.2 [46]. Finally, the suspensions were diluted to an OD of 0.05 and stored for later use.

##### Determination of Inhibition Zones

For this procedure, 1 mL of each of the four bacterial suspensions was added to preprepared sterile LB agar plates. The suspensions were thoroughly spread on the LB agar plates. After the suspensions had settled, the excess liquid was removed using a pipette, and the plates were air-dried [47]. A sterile tweezer was used to pick up a 6 mm diameter sterile filter paper disk, and a pipette was used to add 5 μL of the test essential oil onto the corresponding filter paper disk. The disks were then placed onto the corresponding LB agar medium. The culture plates were inverted and incubated overnight in a 37 °C incubator. After 24 h, the diameters of the inhibition zones were measured. The diameters of the inhibition zones formed by the corresponding essential oil filter paper disks were measured using the cross method. Three parallel experiments were conducted, and the average values were taken to evaluate the bacteriostatic effect of the plant essential oils [48]. DMSO [49] was used as a negative control.

##### Sensitivity Level Test of Essential Oil

For the inhibition zone test, 6 mm-diameter filter paper disks were used. Bacterial sensitivity to the essential oils was determined based on the size of the inhibition zones. The judgment criteria followed the “Standards for the Implementation of Antimicrobial Susceptibility Testing” issued by the CLSI and were as follows: diameter > 20 mm, extremely sensitive; 15–20 mm, highly sensitive; 10–15 mm, moderately sensitive; <10 mm, low sensitivity.

#### 3.3.3. Analysis of LEOs

GC–MS was used for the analysis, and the system was a 7890B-5977A with an HP-5MS chromatographic column (30 m × 250 μm × 0.25 μm).

GC conditions: initial temperature of 40 °C, held for 3 min; increased to 160 °C at a rate of 5 °C/min, then increased to 200 °C at a rate of 10 °C/min, and held for 10 min; injection port temperature of 250 °C; flow rate of 1.0 mL/min; carrier gas was high-purity He; and splitless injection.

MS conditions: electron impact ion source; electron impact energy of 70 eV; ion source temperature of 230 °C; transfer line temperature of 250 °C; scan range of 50–500 *m*/*z*; and MS quadrupole temperature of 150 °C.

The five LEOs were diluted 20-fold, and 0.2 μL of each oil was manually injected. The experiments were repeated thrice, and the final results were expressed as the mean ± standard deviation.

#### 3.3.4. Qualitative Analysis of Volatile Components in LEOs

The volatile components of the plant essential oils were detected and analyzed by using GC–MS to obtain their total ion chromatograms. The NIST/WILEY mass spectral library was used for searching, and the MS, retention index (RI), and literature-reported RI values were combined to identify each compound and determine the volatile substances [14].

RI: the RIs of each component were calculated using the retention times of n-alkanes. The following formula was used to calculate the RI:

$$\left[\mathrm{RI}=100\;\times \left(\mathrm{tR}\left(i\right)\;-\;\mathrm{tR}\left(N\right)\right)\;/\;\left(\mathrm{tR}\left(N+1\right)\;-\;\mathrm{tR}\left(N\right)\right)+\;100N\right]$$

where [tR(i)] is the retention time of the analyte; [tR(N)] and [tR(N + 1)] are the retention times of n-alkanes with carbon numbers N and N + 1, respectively.

The volatile components of the plant essential oils were detected and analyzed using GC–MS to obtain their total ion chromatograms. The NIST/WILEY mass spectral library was used for searching, and the area normalization method was applied for the semiquantitative analysis of the relative content of each component to identify the volatile substances [15].

$$\left[\mathrm{Relative}\;\mathrm{content}\;\left(\%\right)=\mathrm{Peak}\;\mathrm{area}\;\mathrm{of}\;a\;\mathrm{component}\;/\;\mathrm{Total}\;\mathrm{peak}\;\mathrm{area}\;\times \;100\%\right]$$

#### 3.3.5. Aroma Analysis of LEOs

Aroma does not have an exact unit of measurement and is primarily derived from human olfactory perception. It describes the olfactory impression of a particular fragrance tone in perfumes and fragrances and is mostly expressed using descriptive language. Essential oils are extracted from plant materials and exhibit complex and diverse aromas.

Volatile aromatic components are the material basis for the aroma of essential oils [50]. The “ABC” method proposed by Lin Xiangyun in “The Art of Perfumery” was used to quantitatively describe the odors of the volatile components. This system classifies the odors of different volatile components in nature into 26 types, using the initial letter of their representative aroma as an abbreviation. This classification method is the only system to quantitatively describe and distinguish orders quantitatively. By combining the ABC quantitative values of the odor of each fragrance substance with its relative content, a radar chart of the aroma distribution can be drawn.

#### 3.3.6. Data Analysis

SPSS 18.0 (IBM, Armonk, NY, USA) software was used to process the data statistically, and the one-way analysis of variance (ANOVA) was applied to analyze the significance of the difference between groups. The difference was considered significant in the case of *p* < 0.05, and the results were expressed as ±SD.

## 4. Conclusions

In this study, the extraction rate of essential oil by supercritical fluid extraction is 3.72–4.17%. The extraction rate of *Lavender* is the highest, which is 4.17%. Essential oil is basically yellow, and real *Lavender* is lighter in color. The volatile components of five LEOs were identified using GC–MS, and the antibacterial activities of the LEOs against four marine *Vibrio* species were evaluated using disk diffusion and serial dilution methods. The findings suggested that all five LEOs exhibited antibacterial activity against the four tested marine *Vibrio* species. The antibacterial effects of the LEOs exceeded moderate sensitivity. The antibacterial effects of French blue (16 mm), space blue (15.33 mm), wakeful (17.17 mm), and real (15.5 mm) reached highly sensitive standards, particularly against *S. maridflavi*. The essential oil of eye-catching *Lavender* demonstrated good activity against *S. maridflavi*, *S. algae*, *V. alginolyticus*, and *V. harveyi*. For French blue LEO, a total of 29 compounds were identified (96.33%). The main components are linalool (32.72%) and linalyl acetate (31.18%), of which the compounds with content greater than 1% account for 90.43%. For 701 LEO, 29 kinds of volatile substances (99.14%) were identified, among which linalool (35.38%) and linalyl acetate (33.91%) were the main components, and the compounds with content greater than 1% accounted for 93.03%. For space blue LEO, 33 compounds (99.29%) were identified, and the main components were linalool (31%) and linalyl acetate (30.27%), and the compounds with content greater than 1% accounted for 92.24%. For eye-catching LEO, 32 compounds (99.78%) were identified, and the main components were linalool (27.51%) and linalyl acetate (15.58%), and the compounds with content greater than 1% accounted for 92.28%. For true LEO, 27 kinds of compounds (100%) were identified, and the main components were linalyl acetate (40.81%) and linalool (24.6%), and the compounds with content greater than 1% accounted for 92.34%. Different varieties of LEOs possessed similar types of compounds, with the major ones being linalool and linalyl acetate. The main fragrances were the lily of the valley fragrance, aromatic compound fragrance, and herbal fragrance. This study’s findings can provide a reference basis for the prevention and control of Vibrio and the development and application of antibacterial drugs, as well as offer insights into the aroma analysis of the five LEOs.

## Figures and Tables

**Table 1:** Extraction of essential oil from *Lavender*.

Variety	Extraction Yield/%	Color
Eye-catching *Lavender*	4.17 ± 0.11 ^a^	Yellow
Space blue *Lavender*	3.72 ± 0.20 ^b^	Yellow
French blue *Lavender*	3.95 ± 0.16 ^c^	Yellow
701 *Lavender*	3.74 ± 0.18 ^b^	Yellow
True *Lavender*	3.78 ± 0.22 ^b^	Light Yellow

Note: different letters in a column indicate significant differences (*p* < 0.05).

**Table 2:** Sensitivity levels of different Vibrio strains to various essential oils (unit/mm).

Essential Oil Type	*Shewanella maridflavi*	*Shewanella algae*	*Vibrio alginolyticus*	*Vibrio harveyi*
French blue *Lavender*	16.00 ± 0.50	12.75 ± 2.38	13.67 ± 2.36	10.83 ± 1.04
701 *Lavender*	12.50 ± 2.27	12.00 ± 1.47	12.33 ± 1.43	11.00 ± 0.71
Space *Lavender*	15.33 ± 5.51	11.83 ± 2.75	12.33 ± 2.52	13.83 ± 2.75
Eye-catching *Lavender*	17.17 ± 1.70	12.58 ± 0.96	14.83 ± 1.25	13.75 ± 0.54
True *Lavender*	15.50 ± 5.27	10.83 ± 1.44	12.00 ± 3.46	13.17 ± 2.93

**Table 3:** Compound composition of French blue LEO.

NO.	Components	Molecular Formula	RT/min	RI (cal)	RI (ref)	Relative Content/%	CAS	MS
1	*α*-pinene	C_10_H_16_	9.50	930.81	939	0.27 ± 0.02	80-56-8	RI, MS
2	Camphene	C_10_H_16_	9.97	945.75	936	0.27 ± 0.02	79-92-5	RI, MS
3	1-Octen-3-ol	C_8_H_16_O	11.07	986.77	979	0.30 ± 0.02	3391-86-4	RI, MS
4	Myrcene	C_10_H_16_	11.42	991.31	991	0.52 ± 0.03	123-35-3	RI, MS
5	3-Carene	C_10_H_16_	12.00	1009.67	1011	0.45 ± 0.03	13466-78-9	RI, MS
6	Hexyl acetate	C_8_H_16_O_2_	12.17	1023.26	1011	0.27 ± 0.02	142-92-7	RI, MS [20]
7	*p*-Cymene	C_10_H_14_	12.47	1024.58	1026	0.29 ± 0.02	99-87-6	RI, MS
8	*D*-Limonene	C_10_H_16_	12.60	1028.69	1036	0.72 ± 0.05	5989-27-5	RI, MS
9	Eucalyptol	C_10_H_18_O	12.69	1031.63	1037	1.27 ± 0.07	470-82-6	RI, MS
10	*trans*-*β*-Ocimene	C_10_H_16_	12.93	1039.18	1045	4.72 ± 0.28	3779-61-1	RI, MS
11	*β*-Ocimene	C_10_H_16_	13.25	1049.35	1044	2.02 ± 0.12	13877-91-3	RI, MS [21]
12	*α*-terpinene	C_10_H_16_	14.50	1088.69	1085	0.17 ± 0.01	586-63-0	RI, MS
13	Linalool	C_10_H_18_O	14.94	1102.62	1102	32.72 ± 1.96	78-70-6	RI, MS
14	1-Octen-3-yl-acetate	C_10_H_18_O_2_	15.27	1112.95	1108	1.14 ± 0.06	2442-10-6	RI, MS [22]
15	Camphore	C_10_H_16_O	16.24	1143.76	1151	0.48 ± 0.03	464-48-2	RI, MS [23]
16	Isoborneol	C_10_H_18_O	16.92	1165.24	1155	2.26 ± 0.13	124-76-5	RI, MS
17	terpinene-4-ol	C_10_H_18_O	17.24	1175.26	1177	2.56 ± 0.14	562-74-3	RI, MS
18	Cryptone	C_9_H_14_O	17.51	1178.97	1187	0.21 ± 0.01	500-02-7	RI, MS
19	*α*-Terpineol	C_10_H_18_O	17.64	1187.87	1193	1.17 ± 0.07	98-55-5	RI, MS
20	Isobornyl formate	C_11_H_18_O_2_	18.71	1228.59	1239	0.17 ± 0.01	1200-67-5	RI, MS
21	Linalyl acetate	C_12_H_20_O_2_	19.50	1247.94	1253	31.18 ± 1.84	115-95-7	RI, MS
22	Lavandulyl acetate	C_12_H_22_O_3_	20.44	1291.76	1293	6.17 ± 0.38	20777-39-3	RI, MS [24]
23	*α*-Fenchene	C_10_H_16_	22.41	1338.70		0.22 ± 0.02	471-84-1	MS
24	*α*-Copaene	C_15_H_24_	22.91	1379.91	1379	0.4 ± 0.02	3856-25-5	RI, MS
25	*β*-Caryophyllene	C_15_H_24_	23.93	1437.36	1419	3.81 ± 0.24	87-44-5	RI, MS
26	(*E*)-*β*-Famesene	C_15_H_24_	24.78	1465.58	1459	1.41 ± 0.09	18794-84-8	RI, MS
27	*D*-Germacrene	C_15_H_24_	25.48	1488.93	1503	0.72 ± 0.05	18252-44-3	RI, MS [25]
28	*γ*-Cadinene	C_15_H_24_	26.27	1515.41	1513	0.22 ± 0.01	39029-41-9	RI, MS [26]
29	*τ*-Cadinol	C_15_H_26_O	28.98	1605.77		0.22 ± 0.01	5937-11-1	MS
SUM			-	-	-	96.33		

**Table 4:** Compound composition of 701 LEO.

NO.	Components	Molecular Formula	RT/min	RI (cal)	RI (ref)	Relative Content/%	CAS	MS
1	*α*-Pinene	C_10_H_16_	9.50	930.81	939	0.25 ± 0.00	80-56-8	RI, MS
2	Camphene	C_10_H_16_	9.97	945.72	936	0.29 ± 0.00	79-92-5	RI, MS [27]
3	1-Octen-3-ol	C_8_H_16_O	11.07	986.94	979	0.21 ± 0.00	3391-86-4	RI, MS
4	Myrcene	C_10_H_16_	11.42	991.31	991	0.50 ± 0.01	123-35-3	RI, MS
5	3-Carene	C_10_H_16_	11.99	1009.51	1011	0.40 ± 0.00	13466-78-9	RI, MS
6	Hexyl acetate	C_8_H_16_O_2_	12.17	1023.23	1011	0.21 ± 0.00	142-92-7	RI, MS [28]
7	*p*-Cymene	C_10_H_14_	12.47	1024.58	1026	0.41 ± 0.00	99-87-6	RI, MS
8	*D*-Limonene	C_10_H_16_	12.60	1028.69	1036	0.53 ± 0.00	5989-27-5	RI, MS
9	Eucalyptol	C_10_H_18_O	12.69	1031.47	1037	1.51 ± 0.01	470-82-6	RI, MS
10	*trans*-*β*-Ocimene	C_10_H_16_	12.93	1039.18	1045	3.67 ± 0.02	3779-61-1	RI, MS [20]
11	*β*-Ocimene	C_10_H_16_	13.25	1049.35	1044	1.49 ± 0.01	13877-91-3	RI, MS [21]
12	terpinolene	C_10_H_16_	14.53	1089.83	1088	0.19 ± 0.00	586-63-0	RI, MS
13	Linalool	C_10_H_18_O	14.94	1102.78	1102	35.38 ± 0.18	78-70-6	RI, MS
14	1-Octen-3-yl-acetate	C_10_H_18_O_2_	15.27	1112.95	1108	0.62 ± 0.00	2442-10-6	RI, MS [22]
15	Camphore	C_10_H_16_O	16.24	1143.76	1151	0.40 ± 0.00	464-48-2	RI, MS [23]
16	Isoborneol	C_10_H_18_O	16.89	1164.27	1155	1.94 ± 0.01	124-76-5	RI, MS
17	4-Carvomenthenol	C_10_H_18_O	17.23	1175.07	1177	3.71 ± 0.02	562-74-3	RI, MS
18	Cryptone	C_9_H_14_O	17.51	1178.79	1187	0.20 ± 0.00	500-02-7	RI, MS
19	*α*-Terpineol	C_10_H_18_O	17.63	1187.71	1193	1.05 ± 0.01	98-55-5	RI, MS
20	Isobornyl formate	C_11_H_18_O_2_	18.70	1228.42	1239	0.18 ± 0.00	1200-67-5	RI, MS
21	Linalyl acetate	C_14_H_24_O_2_	19.50	1247.94	1253	33.91 ± 0.30	115-95-7	RI, MS
22	Lavandulyl acetate	C_13_H_22_O_3_	20.44	1291.57	1293	3.15 ± 0.01	20777-39-3	RI, MS [24]
23	*α*-Fenchene	C_10_H_16_	22.40	1338.52		0.21 ± 0.00	105-91-9	MS
24	*α*-Copaene	C_12_H_20_O_2_	22.91	1379.91	1379	0.36 ± 0.00	141-12-8	RI, MS
25	*β*-Caryophyllene	C_15_H_24_	23.93	1437.19	1419	5.88 ± 0.04	87-44-5	RI, MS
26	*(E)*-*β*-Famesene	C_15_H_24_	24.78	1465.58	1459	1.34 ± 0.02	18794-84-8	RI, MS
27	*D*-Germacrene	C_15_H_24_	25.47	1488.76	1503	0.68 ± 0.00	18252-44-3	RI, MS [25]
28	*γ*-Cadinene	C_15_H_24_	26.27	1515.24	1513	0.24 ± 0.00	39029-41-9	RI, MS [26]
29	*τ*-Cadinol	C_15_H_26_O	28.97	1605.57		0.23 ± 0.00	5937-11-1	MS
SUM			-	-	-	99.14		

**Table 5:** Compound composition of space blue LEO.

NO.	Components	Molecular Formula	RT/min	RI (cal)	RI (ref)	Relative Content/%	CAS	MS	NO.
1	Camphene	C_10_H_16_	9.97	945.56	936	936	0.46 ± 0.01	79-92-5	RI, MS [27]
2	*β*-Pinene	C_10_H_16_	10.89	974.76	980	980	0.18 ± 0.00	127-91-3	RI, MS
3	1-Octen-3-ol	C_8_H_16_O	11.07	986.77	979	979	0.28 ± 0.01	3391-86-4	RI, MS
4	Myrcene	C_10_H_16_	11.42	991.31	991	991	0.75 ± 0.03	123-35-3	RI, MS
5	3-Carene	C_10_H_16_	11.99	1009.51	1011	1011	1.17 ± 0.02	13466-78-9	RI, MS
6	Hexyl acetate	C_8_H_16_O_2_	12.17	1023.23	1016	1016	0.25 ± 0.01	142-92-7	RI, MS [29]
7	*o*-Cymene	C_10_H_14_	12.39	1021.96	1023	1023	0.22 ± 0.00	527-84-4	RI, MS
8	*p*-Cymene	C_10_H_14_	12.47	1024.58	1026	1026	0.49 ± 0.01	99-87-6	RI, MS
9	*D*-Limonene	C_10_H_16_	12.60	1028.85	1036	1036	1.84 ± 0.04	5989-27-5	RI, MS
10	Eucalyptol	C_10_H_18_O	12.69	1031.63	1037	1037	1.34 ± 0.03	470-82-6	RI, MS [20]
11	*trans*-*β*-Ocimene	C_10_H_16_	12.93	1039.18	1045	1045	4.75 ± 2.15	3779-61-1	RI, MS [21]
12	*β*-Ocimene	C_10_H_16_	13.25	1049.35	1044	1044	1.96 ± 0.05	13877-91-3	RI, MS
13	*α*-terpinene	C_10_H_16_	14.50	1088.69	1085	1085	0.21 ± 0.00	99-86-5	RI, MS
14	Linalool	C_10_H_18_O	14.94	1102.62	1102	1102	31.00 ± 0.81	78-70-6	RI, MS [22]
15	1-Octenyl-3-acetate	C_10_H_18_O_2_	15.27	1112.95	1108	1108	1.04 ± 0.01	2442-10-6	RI, MS [23]
16	Camphore	C_10_H_16_O	16.24	1143.76	1151	1151	0.38 ± 0.01	464-48-2	RI, MS
17	Isoborneol	C_10_H_18_O	16.91	1164.93	1155	1155	2.87 ± 0.09	124-76-5	RI, MS
18	terpinene-4-ol	C_10_H_18_O	17.23	1175.07	1177	1177	1.68 ± 0.07	562-74-3	RI, MS
19	Cryptone	C_9_H_14_O	17.51	1178.79	1187	1187	0.37 ± 0.03	500-02-7	RI, MS [30]
20	*α*-Terpineol	C_10_H_18_O	17.63	1187.71	1193	1193	1.46 ± 0.04	98-55-5	RI, MS
21	Nerol	C_10_H_16_	18.70	1221.48	1228	1228	0.24 ± 0.00	106-25-2	RI, MS [31]
22	Hexyl 2-methylbutyrate	C_11_H_22_O_2_	19.04	1239.85	1238	1238	0.17 ± 0.01	10032-15-2	RI, MS
23	Linalyl acetate	C_12_H_20_O_2_	19.50	1258.85	1253	1253	30.27 ± 0.63	115-95-7	RI, MS
24	Bornyl acetate	C_12_H_20_O_2_	20.34	1288.55	1292	1292	0.25 ± 0.01	76-49-3	RI, MS [31]
25	Lavandulyl acetate	C_12_H_22_O_3_	20.44	1292.25	1293	1293	6.51 ± 0.18	20777-39-3	RI, MS [24]
26	Neryl acetate	C_12_H_20_O_2_	22.41	1362.20	1366	1366	0.35 ± 0.01	141-12-8	RI, MS
27	*α*-Copaene	C_15_H_24_	22.91	1403.27	1379	1379	0.59 ± 0.04	3856-25-5	RI, MS
28	*β*-Caryophyllene	C_15_H_24_	23.93	1437.36	1431	1431	5.04 ± 0.15	87-44-5	RI, MS
29	*trans*-*α*-bergamotene	C_15_H_24_	24.29	1449.30	1436	1436	0.24 ± 0.01	13474-59-4	RI, MS
30	(*E*)-*β*-Famesene	C_15_H_24_	24.78	1465.74	1459	1459	1.31 ± 0.03	18794-84-8	RI, MS
31	D-Germacrene	C_15_H_24_	25.48	1488.93	1503	1503	0.86 ± 0.02	23986-74-5	RI, MS [25]
32	*γ*-Cadinene	C_15_H_24_	26.27	1515.41	1513	1513	0.42 ± 0.01	39029-41-9	RI, MS
33	*τ*-Cadinol	C_15_H_26_O	28.98	1605.74			0.34 ± 0.01	5937-11-1	MS
SUM			-	-		-	99.29		

**Table 6:** Compound composition of eye-catching LEO.

NO.	Components	Molecular Formula	RT/min	RI (cal)	RI (ref)	Relative Content/%	CAS	MS
1	*α*-pinene	C_10_H_16_	9.50	930.81	939	1.18 ± 0.00	80-56-8	RI, MS
2	Camphene	C_10_H_16_	9.97	945.75	936	0.72 ± 0.00	79-92-5	RI, MS [27]
3	*α*-Sabinene	C_10_H_16_	10.81	972.29	970	0.43 ± 0.00	3387-41-5	RI, MS
4	*β*-Pinene	C_10_H_16_	10.90	974.91	980	1.35 ± 0.01	127-91-3	RI, MS
5	1-Octen-3-ol	C_8_H_16_O	11.07	986.97	979	0.23 ± 0.01	3391-86-4	RI, MS
6	3-Octanone	C_8_H_16_O	11.30	994.33	986	0.20 ± 0.00	106-68-3	RI, MS [32]
7	Myrcene	C_10_H_16_	11.42	991.31	991	0.79 ± 0.02	123-35-3	RI, MS
8	3-Carene	C_10_H_16_	12.20	1016.24	1018	0.19 ± 0.00	13466-78-9	RI, MS [33]
9	*p*-Cymene	C_10_H_14_	12.47	1024.74	1026	0.21 ± 0.00	99-87-6	RI, MS
10	*D*-Limonene	C_10_H_16_	12.60	1028.85	1036	1.57 ± 0.01	5989-27-5	RI, MS
11	Eucalyptol	C_10_H_18_O	12.70	1031.97	1037	18.16 ± 0.06	470-82-6	RI, MS
12	trans-*β*-Ocimene	C_10_H_16_	12.93	1039.18	1045	1.85 ± 0.01	3779-61-1	RI, MS [20]
13	*β*-Ocimene	C_10_H_16_	13.26	1049.51	1044	2.88 ± 0.01	13877-91-3	RI, MS [21]
14	*γ*-Terpinene	C_10_H_16_	13.57	1059.49	1063	0.19 ± 0.00	99-85-4	RI, MS
15	*α*-terpinene	C_10_H_16_	14.50	1088.69	1085	0.54 ± 0.01	99-86-5	RI, MS
16	Linalool	C_10_H_18_O	14.94	1102.78	1102	27.51 ± 0.08	78-70-6	RI, MS
17	1-Octen-3-yl-acetate	C_10_H_18_O_2_	15.27	1113.11	1108	0.19 ± 0.05	2442-10-6	RI, MS [22]
18	Camphore	C_10_H_16_O	16.25	1144.11	1151	11.87 ± 0.05	21368-68-3	RI, MS [23]
19	Isoborneol	C_10_H_18_O	16.89	1164.42	1155	3.29 ± 0.03	124-76-5	RI, MS
20	terpinene-4-ol	C_10_H_18_O	17.24	1175.26	1177	0.85 ± 0.02	562-74-3	RI, MS
21	*α*-Terpineol	C_10_H_18_O	17.64	1187.87	1193	1.66 ± 0.02	98-55-5	RI, MS
22	Linalyl acetate	C_12_H_20_O_2_	19.49	1258.50	1253	15.58 ± 0.04	115-95-7	RI, MS
23	Lavandulyl acetate	C_12_H_20_O_2_	20.44	1292.07	1293	1.46 ± 0.01	20777-39-3	RI, MS [24]
24	(*E*)-Hexyl 2-methylbut-2-enoate	C_11_H_20_O_2_	21.52	1322.51	1331	0.15 ± 0.00	16930-96-4	RI, MS
25	*α*-Fenchene	C_10_H_16_	22.41	1338.70		0.25 ± 0.17	471-84-1	MS
26	*β*-Caryophyllene	C_15_H_24_	23.93	1437.36	1419	3.92 ± 0.03	87-44-5	RI, MS
27	trans-*α*-bergamotene	C_15_H_24_	24.29	1449.47	1436	0.28 ± 0.01	13474-59-4	RI, MS [34]
28	(*E*)-*β*-Famesene	C_15_H_24_	24.78	1465.74	1459	0.55 ± 0.01	18794-84-8	RI, MS
29	*D*-Germacrene	C_15_H_24_	25.48	1489.09	1503	0.70 ± 0.01	23986-74-5	RI, MS [25]
30	Neryl propionate	C_13_H_22_O_2_	26.08	1504.91	1515	0.38 ± 0.01	105-91-9	RI, MS
31	*γ*-Cadinene	C_15_H_24_	26.28	1515.58	1513	0.32 ± 0.00	39029-41-9	RI, MS [26]
32	*τ*-Cadinol	C_15_H_26_O	28.98	1605.94		0.33 ± 0.01	5937-11-1	MS
SUM			-	-	-	99.78		

**Table 7:** Compound composition of true LEO.

NO.	Components	Molecular Formula	RT/min	RI (cal)	RI (ref)	Relative Content/%	CAS	MS
1	*α*-Pinene	C_10_H_16_	9.50	930.65	939	0.23 ± 0.00	80-56-8	RI, MS
2	1-Octen-3-ol	C_8_H_16_O	11.07	986.77	979	0.21 ± 0.00	3391-86-4	RI, MS
3	3-Octanone	C_8_H_16_O	11.28	993.80	986	1.17 ± 0.01	106-68-3	RI, MS [32]
4	*β*-Myrcene	C_10_H_16_	11.41	991.15	991	0.28 ± 0.01	123-35-3	RI, MS
5	3-Octanol	C_8_H_18_O	11.59	1004.06	997	0.27 ± 0.00	589-98-0	RI, MS
6	Hexyl acetate	C_8_H_16_O_2_	12.17	1023.06	1011	0.27 ± 0.00	142-92-7	RI, MS [28]
7	*p*-Cymene	C_10_H_14_	12.47	1024.58	1026	0.26 ± 0.00	99-87-6	RI, MS
8	*D*-Limonene	C_10_H_16_	12.60	1028.69	1036	0.95 ± 0.01	5989-27-5	RI, MS
9	Eucalyptol	C_10_H_18_O	12.69	1031.47	1037	0.90 ± 0.01	470-82-6	RI, MS
10	*trans*-*β*-Ocimene	C_10_H_16_	12.93	1039.02	1045	3.18 ± 0.03	3779-61-1	RI, MS [20]
11	*β*-Ocimene	C_10_H_16_	13.25	1049.19	1044	1.57 ± 0.01	13877-91-3	RI, MS
12	Linalol	C_10_H_18_O	14.93	1102.46	1102	24.60 ± 0.19	78-70-6	RI, MS
13	1-Octenyl-3-acetate	C_10_H_18_O_2_	15.26	1112.80	1108	0.83 ± 0.01	2442-10-6	RI, MS
14	Camphore	C_10_H_16_O	16.24	1143.76	1151	0.24 ± 0.00	21368-68-3	RI, MS
15	Isoborneol	C_10_H_18_O	16.92	1165.09	1155	1.63 ± 0.00	124-76-5	RI, MS
16	terpinene-4-ol	C_10_H_18_O	17.23	1175.07	1177	3.70 ± 0.03	562-74-3	RI, MS
17	Cryptone	C_9_H_14_O	17.50	1178.64	1187	0.25 ± 0.02	500-02-7	RI, MS
18	*α*-Terpineol	C_10_H_18_O	17.63	1187.71	1193	0.42 ± 0.01	10482-56-1	RI, MS
19	Linalyl acetate	C_12_H_20_O_2_	19.52	1248.58	1253	40.81 ± 0.21	115-95-7	RI, MS
20	Lavandulyl acetate	C_12_H_20_O_2_	20.44	1291.76	1293	4.14 ± 0.01	20777-39-3	RI, MS [24]
21	*β*-Caryophyllene	C_15_H_24_	23.93	1437.36	1419	5.83 ± 0.01	87-44-5	RI, MS
22	*trans*-2-Hydroxycinnamic acid	C_9_H_8_O_3_	24.34	1388.87		1.62 ± 0.01	614-60-8	MS
23	(*E*)-*β*-Famesene	C_15_H_24_	24.78	1465.74	1459	4.09 ± 0.02	18794-84-8	RI, MS
24	*D*-Germacrene	C_15_H_24_	25.47	1488.76	1503	0.91 ± 0.01	23986-74-5	RI, MS [25]
25	Caryophyllene oxide	C_15_H_24_O	27.89	1569.58		0.81 ± 0.01	1139-30-6	MS
26	*τ*-Cadinol	C_15_H_26_O	28.98	1605.77		0.22 ± 0.00	5937-11-1	MS
27	Herniarin	C_10_H_8_O_3_	30.35	1589.70		0.67 ± 0.03	531-59-9	MS
SUM			-	-	-	100		

## Data Availability

Data are contained within the article.
